# Effects of vitamin D supplementation on metabolic parameters in women with polycystic ovary syndrome: a randomized controlled trial

**DOI:** 10.1186/s13048-024-01473-6

**Published:** 2024-07-16

**Authors:** Xinling Wen, Li Wang, Fen Li, Xuewen Yu

**Affiliations:** 1 Department of Anesthesiology and Operation, The First Affiliated Hospital of Xi’an Jiaotong University, Xi’an, Shaanxi China; 2 Department of Gynecology and Obstetrics, The First Affiliated Hospital of Xi’an Jiaotong University, Xi’an, Shaanxi China

**Keywords:** Vitamin D, Polycystic ovary syndrome, Vitamin D deficiency, Metabolic diseases, Insulin resistance, Obesity

## Abstract

**Objective:**

The aim of this study was to explore the effects of vitamin D supplementation on metabolic parameters in women with polycystic ovary syndrome (PCOS).

**Methods:**

A total of 60 PCOS women with vitamin D deficiency or insufficiency were enrolled in this randomized controlled trial. Participants were randomized to vitamin D group (2000 IU/day) or control group. The observational parameters were measured at baseline and after treatment, including body mass index (BMI), waist to hip ratio (WHR), oral glucose tolerance test (OGTT) and insulin release test, and lipid metabolism parameters.

**Results:**

The serum 25(OH)D concentrations at different time points after vitamin D supplementation were significantly higher than that in control group (*P* < 0.05). The BMI, WHR, insulin concentrations, homeostasis model assessment of insulin resistance (HOMA-IR), triglycerides (TG), total cholesterol (TC) and low-density lipoprotein cholesterol (LDL-C) concentrations in women of Vitamin D group after 12 weeks of treatment were significantly lower than that in women of control group (*P <* 0.05). The serum insulin concentrations and HOMA-IR at different time points of OGTT, serum TG, TC and LDL-C concentrations in women of vitamin D group (obesity) were significantly lower compared with control group (obesity) (*P <* 0.05). The BMI, WHR, TG, TC and LDL-C concentration in women of vitamin D group (IR) were significantly lower compared with control group (IR) (*P <* 0.05). No significant difference was observed in metabolic parameters between vitamin D group (non-obesity) and control group (non-obesity) (*P* > 0.05), and these differences of metabolic parameters were also not observed between vitamin D group (non-IR) and control group (non-IR) (*P* > 0.05).

**Conclusion:**

Vitamin D supplementation had beneficial effects on metabolic parameters in PCOS women, especially in women with obesity or insulin resistance.

## Introduction

Polycystic ovary syndrome (PCOS) is the most common reproductive, endocrine, and metabolic disorders affecting 6–10% of reproductive-age women all over the world [1]. Although the pathogenesis of PCOS has not been fully elucidated, it is thought that some multifactorial causes, including genetic factors, may play a role in its etiology [2]. Women with PCOS are often identified with symptoms of menstrual disorder, infertility, hirsutism, acne and metabolic disorder. Previous studies have confirmed that PCOS is associated with metabolic disorders, such as type 2 diabetes (T2DM), dyslipidemia, cardiovascular disease (CVD), atherosclerosis, metabolic syndrome (MS), nonalcoholic fatty liver disease (NAFLD), and ultimately cirrhosis [3, 4]. In recent years, these metabolic problems in PCOS women attracted the attention of scholars worldwide. A systematic review and meta-analysis showed that 40–50% of women suffering from PCOS have impaired glucose tolerance (IGT), insulin resistance (IR), compensatory hyperinsulinaemia (HI), and that approximately 10% of these women will develop T2DM [5]. Additionally, PCOS women are prone to dyslipidemia, including higher levels of triglycerides (TG), total cholesterol (TC) and low-density lipoprotein cholesterol (LDL-C) than healthy women [6–8].

The molecular action of vitamin D is involved in maintaining the normal resting levels of reactive oxygen species (ROS) and Ca^2+^, not only in pancreatic β-cells, but also in insulin responsive tissues. Vitamin D prevents epigenetic alterations associated with IR, and vitamin D deficiency is one of the factors accelerating IR formation [9]. Moreover, studies have confirmed that vitamin D supplementation reduce the level of metabolic parameters such as TC, LDL-C, TG, glycated hemoglobin (HbA1c), as well as decreases homeostasis model assessment of insulin resistance (HOMA-IR) in patients with T2DM [10, 11].

Multiple epidemiological data worldwide have confirmed that vitamin D deficiency and insufficiency are common in women with PCOS, especially in those with obesity or IR [12–14]. Obesity decreased the fertility of women in PCOS especially due to cycle cancellation [15]. A recent meta-analysis displayed that 67–85% PCOS women had vitamin D deficiency [16]. However, the effects of vitamin D supplementation on metabolic parameters of women with PCOS are controversial. Some of studies shown beneficial effects of vitamin D supplementation on glucose metabolism, IR, dyslipidemia, cardiovascular risk factors, liver markers, metabolic profiles and NAFLD [17, 18]. Nevertheless, other studies suggested that vitamin D supplementation had no significant effect on metabolic and endocrine parameters in PCOS women [19].

In recent years, the therapeutic schedule for PCOS women are improvement of patient’s clinical symptoms and treatment of infertility. However, this may not be completely effective in preventing long-term complications, such as metabolic disorders, DM, dyslipidemia, CVD, NAFLD and endometrial carcinoma. These long-term complications can seriously affect women’s health and quality of life. The aim of this study was to explore the effects of vitamin D supplementation on metabolic parameters in women with PCOS.

## Materials and methods

### Study design and participants

We conducted a randomized controlled trial (RCT) study on PCOS patients with vitamin D deficiency or insufficiency at the First Affiliated Hospital of Xi’an Jiaotong University from January 2021 to June 2023. All participants gave written informed consent on the basis of procedures granted by the Ethics Committee of The First Affiliated Hospital of Medical College of Xi’an Jiaotong University (XJTU1AF2021LSK-207). This study has been registered on China’s clinical trials registration: www.chictr.org.cn (ChiCTR2100048736).

Sixty-eight PCOS women were enrolled in this study. All patients were aged 21–34 years. The diagnostic criteria for PCOS we used were the Rotterdam criteria: chronic ovulatory dysfunction, clinical manifestations or biochemical evidence of hyperandrogenism, and polycystic ovarian morphology (PCOM) [20]. PCOS could be diagnosed when any two of these three criteria were presented. Hyperandrogenism was identified as either clinical manifestations or laboratory evidence. Laboratory evidence was defined as an abnormally increased testosterone level. Clinical manifestations included hirsutism or acne. Hirsutism was defined as a modified Ferriman-Gallwey score of more than 3 at the time of physical examination. PCOM was identified as the presence of 12 or more follicles in unilateral ovary or bilateral ovaries measuring 2–9 mm in diameter, and/or ovarian volume ≥ 10 ml. Ovarian volume = 0.5 × length diameter × transverse diameter × anteroposterior diameter. Participants who were diagnosed with tumor secreting androgens, thyroid dysfunction, hyperprolactinemia, hypothalamic amenorrhea, Cushing’s syndrome, congenital adrenal hyperplasia (CAH), premature ovarian insufficiency (POI), premature ovarian failure (POF), hypercalcemia, malabsorption disorders and diabetes mellitus were excluded. In addition, participants who used corticosteroids, hypolipidemic agents, calcium supplements, or any other drugs known to affect vitamin D metabolism were also excluded from the study.

The baseline data of PCOS women were collected by questionnaire in a face-to-face interview, such as age, body mass index (BMI), waist-to-hip ratio (WHR), blood pressure, marital status, gravidity, parity, employment status, family history and outdoor exercise. BMI ≥ 30.0 kg/m^2^ was defined as obesity [21].

**Fig. 1:**
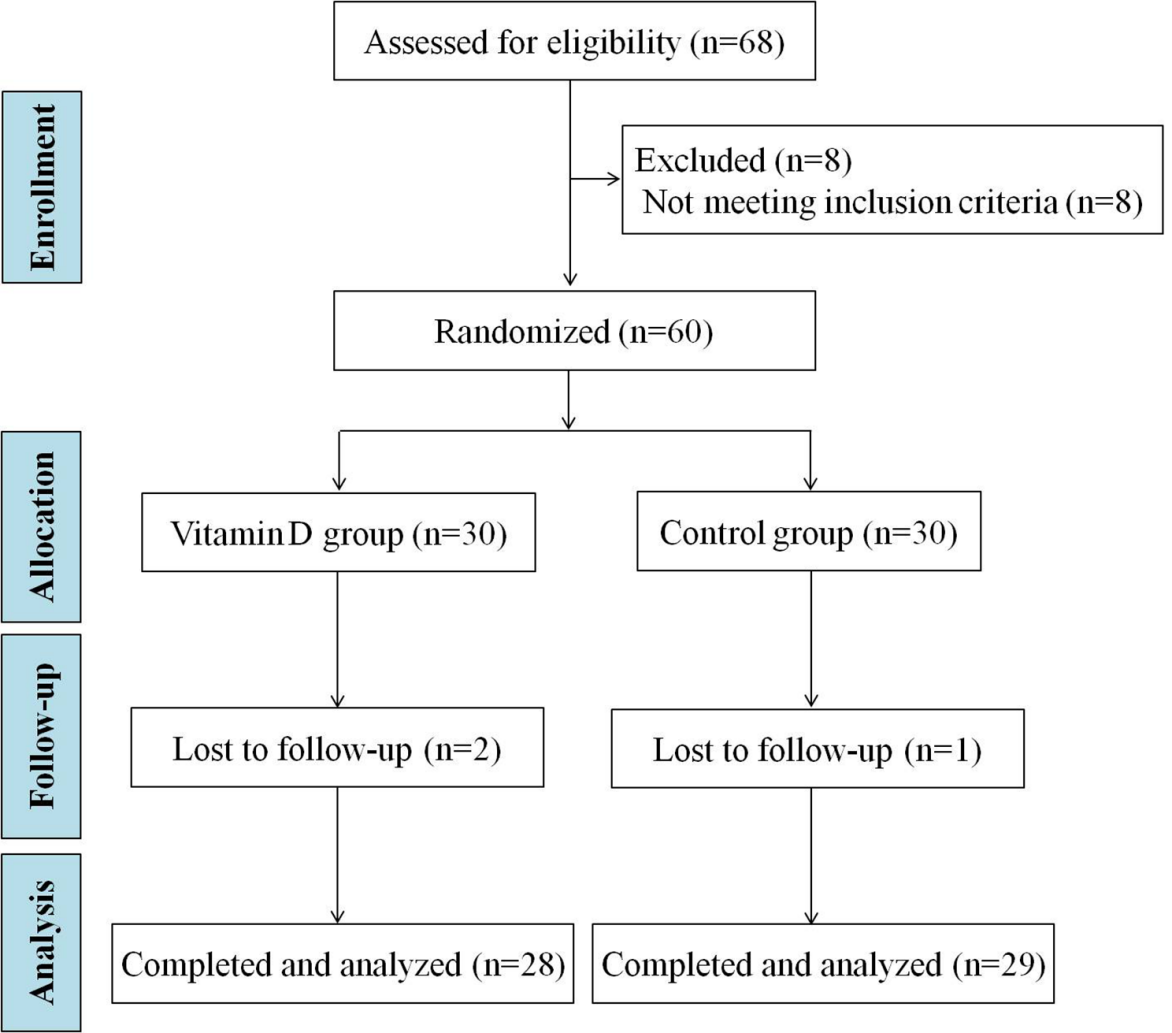
Flow chart of study participation

Sixty-eight PCOS women were enrolled initially in this RCT study, 8 women with normal serum vitamin D level were excluded. Then, sixty PCOS women with vitamin D deficiency or insufficiency were randomly divided into vitamin D group or control group, and 30 participants in each group. Two participants in the vitamin D group and 1 participant in the control group were lost to follow-up. Finally, 57 participants (vitamin D group, *n* = 28. control group, *n* = 29) completed the 12 weeks study period (Fig. 1).

### Measurement of serum vitamin D

25-hydroxyvitamin D3 [25(OH)D] is the primary circulating form of vitamin D, which is the most abundant vitamin D metabolite and considered as the best parameter of vitamin D status within the human body. Therefore, serum 25(OH)D was detected by chemiluminescence method. According to the Institute of Medicine (IOM) and World Health Organization (WHO), vitamin D deficiency was defined as serum 25 (OH) D concentration lower than 20 ng/mL, and vitamin D insufficiency was defined as a concentration of 20–*<*30 ng/mL. Serum 25(OH)D concentration of 30–50 ng/mL were regarded as normal [22].

### Detection of biochemical indicators

All biochemical indicators in this study were detected in the clinical laboratory of our hospital. The basic sex hormone concentrations and anti-Müllerian hormone (AMH) concentration were tested during 2–4 days of natural menstrual cycle or progesterone withdrawal bleeding using chemiluminescence method. Sex hormone included follicle-stimulating hormone (FSH), luteinizing hormone (LH), prolactin (PRL), estradiol (E_2_), progesterone (P) and testosterone (T). All participants were given 83 g oral glucose for oral glucose tolerance test (OGTT) and insulin release test. The blood glucose was detected by hexokinase method. Serum insulin level was detected through radioimmunoassay. HOMA-IR was calculated to evaluate IR. HOMA-IR = [fasting glucose (mmol/L) × fasting insulin (mIU/L)]/22.5. A HOMA-IR > 3.0 was defined as IR, and an insulin level after oral glucose powder intake 10 times higher than the fasting level was also defined as IR [23]. At the same time, the blood lipids were detected using automatic biochemical analyzer, including TC, TG, LDL-C and high-density lipoprotein cholesterol (HDL-C).

### Sample size

Sample size was calculated provided that serum 25(OH)D concentration and glucose metabolism and lipid metabolism indicators were the primary outcomes. According to the method reported in previous literatures, sample size was calculated with the following parameters: probability of type one error (α) of 0.05 and type two error (β) of 0.20 (power = 80%), difference between two means to be detected was 0.52, expected background standard deviation was 1. Based on this, we needed 27 participants in each group. Considering a loss to follow-up of 3 participants per group, we needed to have 30 participants in each group.

### Randomization and intervention

All participants were randomly divided into vitamin D group or control group by means of computer-generated random numbers. Participants in vitamin D group received basic treatment combined with vitamin D supplementation (2000 IU/day). Basic treatment included a proper diet and aerobic exercise outdoors at least three times per week. Each aerobic exercise lasted at least 30 min. Participants in control group were only given basic treatment.

### Outcome measures

The primary outcomes were the serum 25(OH)D concentration, glucose metabolism, insulin concentration and lipid metabolism indicators. The secondary outcomes were general metabolic parameters, including BMI, WHR and blood pressure.

### Statistical analysis

Statistical analyses were performed using SPSS version 20.0 (IBM, Armonk, NY, USA). The Kolmogorov–Smirnov test was used to check the normal distribution prior to statistical tests. For normally distributed variables, the continuous variables were represented as mean ± standard deviation and were analyzed by Student’s t test, whereas the Mann–Whitney U test was used to analyze non-normally distributed data. Differences in dichotomous outcomes were given as number and percentage (%), which were compared by chi-square test. *P <* 0.05 was considered statistically significant.

## Results

### Baseline data

Table 1 shows the baseline data of women between the two groups. No significant difference was found when comparing the baseline data between the two groups (*P* > 0.05).

**Table 1:** Baseline data of women between the two groups

Characteristics	Vitamin D group (*n* = 28)	Control group (*n* = 29)	*P*-value ^a^
Age (years)	26.7 ± 9.3	25.3 ± 8.6	0.764
BMI (kg/m^2^)	25.2 ± 7.4	24.9 ± 7.9	0.812
WHR	0.9 ± 0.3	0.9 ± 0.4	0.978
SBP (mmHg)	109.5 ± 12.3	107.6 ± 11.9	0.714
DBP (mmHg)	78.4 ± 6.5	75.1 ± 7.0	0.256
Marital status			0.705
Single	12 (42.9)	11 (37.9)	
Married	16 (57.1)	18 (62.1)	
Gravidity (number)	3.6 ± 1.2	3.3 ± 1.1	0.798
Parity (number)	1.3 ± 0.6	1.2 ± 0.5	0.609
Employment status			0.561
Working	23 (82.1)	22 (75.9)	
Non-working	5 (17.9)	7 (24.1)	
Family history			
DM	3 (10.7)	4 (13.8)	0.723
CVD	4 (14.3)	4 (13.8)	0.957
Thyroid diseases	2 (7.1)	3 (10.3)	0.669
Outdoor exercise			0.561
Never	5 (17.9)	7 (24.1)	
≥ 1 times daily	23 (82.1)	22 (75.9)	
Basal concentration			
FSH (mIU/mL)	6.1 ± 1.5	6.9 ± 1.6	0.568
LH (mIU/mL)	15.6 ± 4.9	17.1 ± 4.5	0.609
PRL (ng/mL)	14.8 ± 6.2	15.3 ± 5.9	0.544
E_2_ (pmol/L)	129.3 ± 17.5	136.4 ± 15.8	0.312
T (nmol/L)	2.1 ± 0.6	1.9 ± 0.5	0.377
P (nmol/L)	1.2 ± 0.4	1.0 ± 0.3	0.459
AMH (ng/mL)	5.6 ± 1.5	5.3 ± 1.7	0.780
Serum 25(OH)D detection time			0.974
Spring	8 (28.6)	9 (31.0)	
Summer	9 (32.1)	9 (31.0)	
Autumn	5 (17.9)	6 (20.7)	
Winter	6 (21.4)	5 (17.2)	
Serum 25(OH)D concentrations (ng/mL)	12.3 ± 4.6	13.0 ± 4.9	0.517
Vitamin D status			0.786
Deficiency	23 (82.1)	23 (79.3)	
Insufficiency	5 (17.9)	6 (20.7)	
BGC (mmol/L)			
Fasting	4.1 ± 0.9	4.2 ± 0.8	0.879
1 h after OGTT	9.2 ± 1.3	9.8 ± 1.4	0.776
2 h after OGTT	6.7 ± 1.0	7.2 ± 1.1	0.564
3 h after OGTT	4.3 ± 0.8	4.2 ± 0.7	0.902
Insulin level (mIU/L)			
Fasting	27.7 ± 5.8	29.4 ± 8.9	0.213
1 h after OGTT	312.5 ± 56.4	297.6 ± 49.0	0.204
2 h after OGTT	206.8 ± 44.3	187.5 ± 40.2	0.290
3 h after OGTT	61.5 ± 10.4	58.3 ± 11.2	0.576
HOMA IR			
Fasting	5.0 ± 0.6	5.5 ± 0.7	0.145
1 h after OGTT	127.8 ± 19.7	130.1 ± 21.6	0.690
2 h after OGTT	61.2 ± 15.2	60.1 ± 13.9	0.887
3 h after OGTT	11.8 ± 3.9	10.9 ± 3.2	0.468
TG (mmol/L)	1.9 ± 0.3	1.7 ± 0.4	0.443
TC (mmol/L)	5.6 ± 1.4	5.8 ± 1.3	0.865
LDL-C (mmol/L)	3.4 ± 1.1	3.6 ± 1.1	0.409
HDL-C (mmol/L)	1.3 ± 0.5	1.5 ± 0.6	0.357

^a^ T-test or chi-square test. Data given as mean ± SD or number (%)

SBP: systolic blood pressure, DBP: diastolic blood pressure. BGC: blood glucose concentration

### Serum vitamin D concentrations

The data in Table 2 illustrate that the serum 25(OH)D concentrations at different time points after vitamin D supplementation were significantly higher than that in control group (*P* < 0.05). In addition, with the extension of treatment time, the serum 25(OH)D concentration gradually increased.

**Table 2:** The serum 25(OH)D concentrations at baseline and different times after treatment (ng/mL)

	Vitamin D group (*n* = 28)	Control group (*n* = 29)	*P*-value ^a^
Baseline	12.3 ± 4.6	13.0 ± 4.9	0.517
4 weeks after treatment	21.2 ± 5.4	14.8 ± 5.3	0.032
8 weeks after treatment	32.8 ± 6.9	15.9 ± 6.2	0.021
12 weeks after treatment	44.2 ± 9.3	13.6 ± 5.9	0.008

^a^ T-test. Data given as mean ± SD

### Effects of vitamin D supplementation on general metabolic parameters

Table 3 demonstrates the general metabolic parameters between the two groups. The data reveal that BMI and WHR of women in Vitamin D group 12 weeks of treatment were significantly reduced compared with baseline (*P <* 0.05). In addition, the above parameters 12 weeks of treatment in vitamin D group were also significant lower than that in women of control group (*P* = 0.045, *P* = 0.048). However, no significant difference was found when comparing SBP and DPB between baseline and different times after treatment (*P* > 0.05). These significant differences after treatment were also not observed between vitamin D group and control group (*P* = 0.675, *P* = 0.326).

**Table 3:** The effects on general metabolic parameters between the two groups

Characteristics	Vitamin D group (*n* = 28)					Control group (*n* = 29)			
	Baseline	12 weeks	Change	*P*-value ^a^		Baseline	12 weeks	Change	*P*-value ^a^
BMI (kg/m^2^)	25.2 ± 7.4	23.1 ± 6.5^▲^	-2.1 ± 0.9	0.041		24.9 ± 7.9	24.6 ± 7.4	-0.3 ± 0.1	0.219
WHR	0.9 ± 0.3	0.7 ± 0.3^▲^	-0.2 ± 0.1	0.043		0.9 ± 0.4	0.8 ± 0.3	-0.1 ± 0.1	0.307
SBP (mm Hg)	109.5 ± 12.3	106.4 ± 11.5	-3.1 ± 0.9	0.431		107.6 ± 11.9	105.9 ± 10.3	-1.7 ± 0.8	0.685
DPB (mm Hg)	78.4 ± 6.5	76.7 ± 6.2	-1.7 ± 0.6	0.309		75.1 ± 7.0	73.8 ± 6.5	-1.3 ± 0.7	0.443

^a^ T-test. Data given as mean ± SD

^▲^Vitamin D group vs. Control group after 12 weeks of treatment, *P <* 0.05

### Effects of vitamin D supplementation on biochemical metabolic parameters

**Fig. 2:**
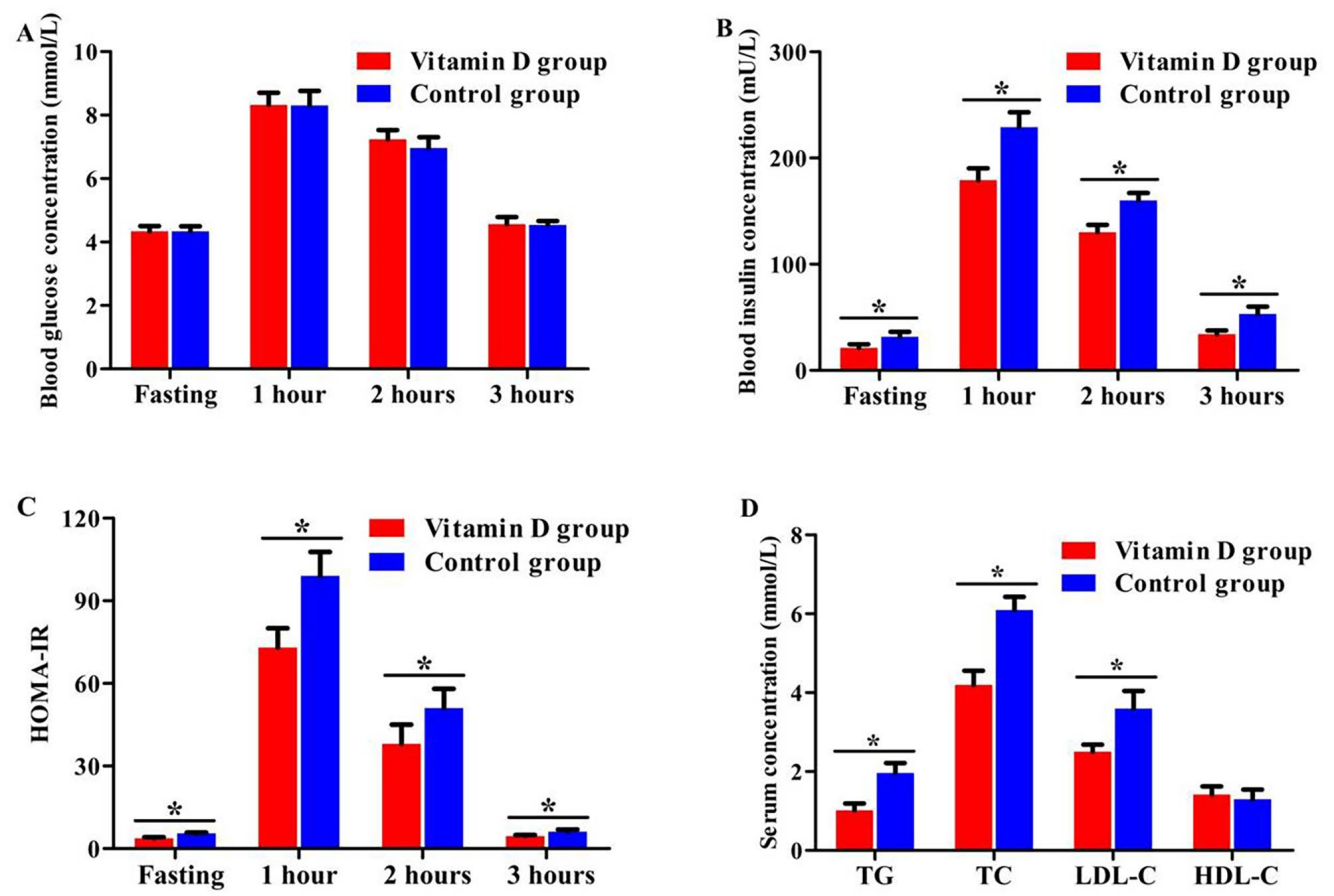
Comparison of biochemical metabolic parameters between the two groups (**A**: Blood glucose concentrations at different time points of OGTT, **B**: Serum insulin concentrations at different time points of OGTT, **C**: HOMA-IR at different time points of OGTT, **D**: Blood lipid parameters. ^*^P < 0.05)

The biochemical metabolic parameters at 12 weeks after treatment were detected between the two groups. The serum insulin concentrations at fasting, 1 h, 2 h and 3 h after OGTT in women of Vitamin D group were significantly lower than that in control group (*P* = 0.046, *P* = 0.029, *P* = 0.035, *P* = 0.041). The HOMA-IR at the above time point were also lower compared with control group (*P* = 0.048, *P* = 0.021, *P* = 0.033, *P* = 0.047). Moreover, the TG, TC and LDL-C concentrations in women of vitamin D group were significantly lower than that in control group (*P* = 0.031, *P* = 0.027, *P* = 0.034). However, no significant difference was found when comparing blood glucose concentrations at different time points of OGTT and HDL-C concentration between the two groups (*P* = 0.342–0.835) (Fig. 2).

**Fig. 3:**
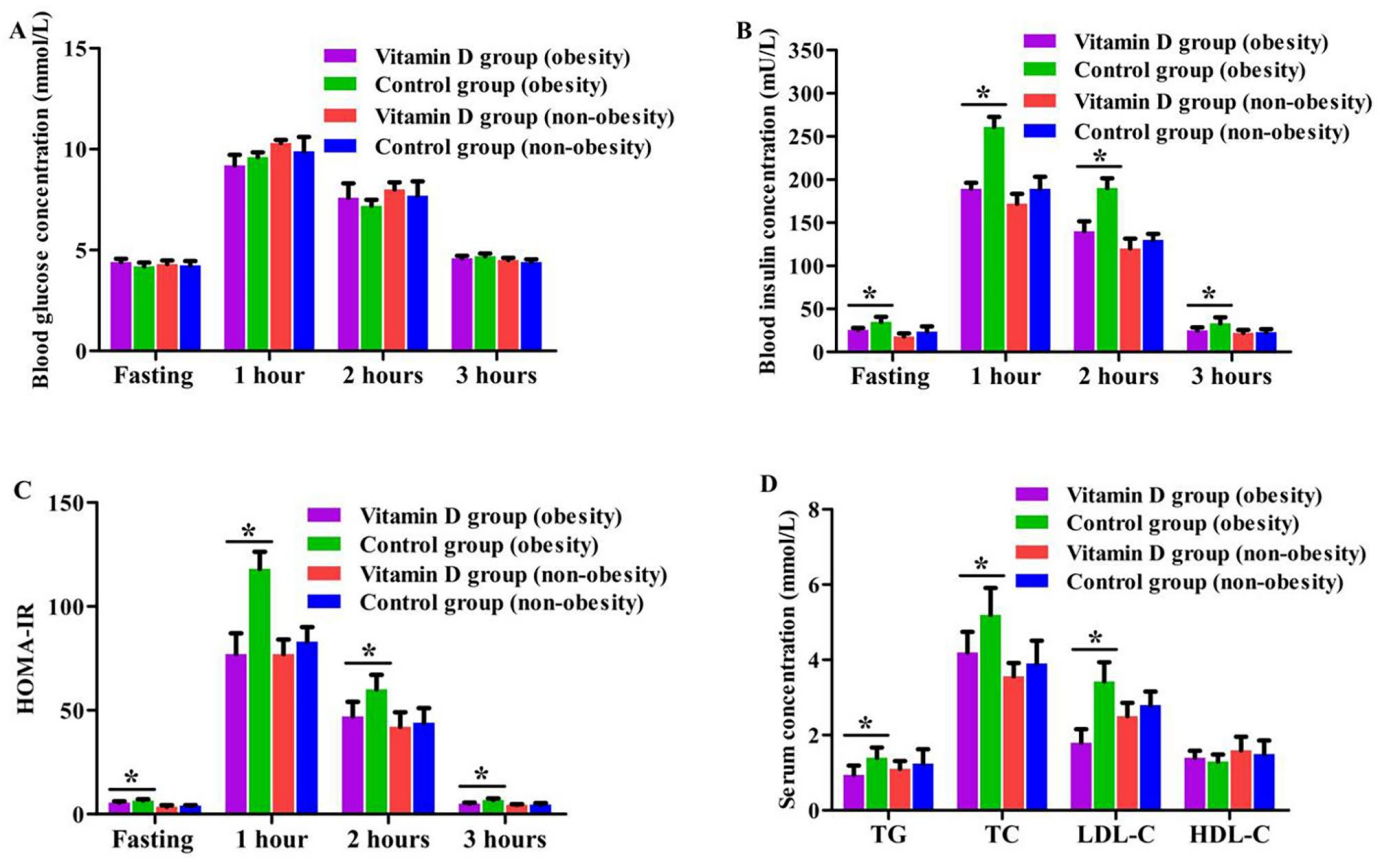
Comparison of biochemical metabolic parameters among the four groups (**A**: Blood glucose concentrations at different time points of OGTT, **B**: Blood insulin concentrations at different time points of OGTT, **C**: HOMA-IR at different time points of OGTT, **D**: Blood lipid parameters. ^*^P < 0.05)

All participants were divided into four groups according to BMI: vitamin D group (obesity) (*n* = 8), vitamin D group (non-obesity) (*n* = 20), control group (obesity) (*n* = 10), control group (non-obesity) (*n* = 19). The biochemical metabolic parameters at baseline and 12 weeks after treatment were detected among the four groups. The serum insulin concentrations at fasting, 1 h, 2 h and 3 h after OGTT in women of vitamin D group (obesity) were significantly lower than that in control group (obesity) (*P* = 0.041, *P* = 0.022, *P* = 0.030, *P* = 0.043). Additionally, the HOMA-IR at the above time point OGTT, TG concentration, TC concentration and LDL-C concentration were also significantly reduced in vitamin D group (obesity) compared with women in control group (obesity) (*P* = 0.018–0.046). Nevertheless, no significant difference was observed of the biochemical metabolic parameters between vitamin D group (non-obesity) and control group (non-obesity) (*P* = 0.317–0.806) (Fig. 3).

**Fig. 4:**
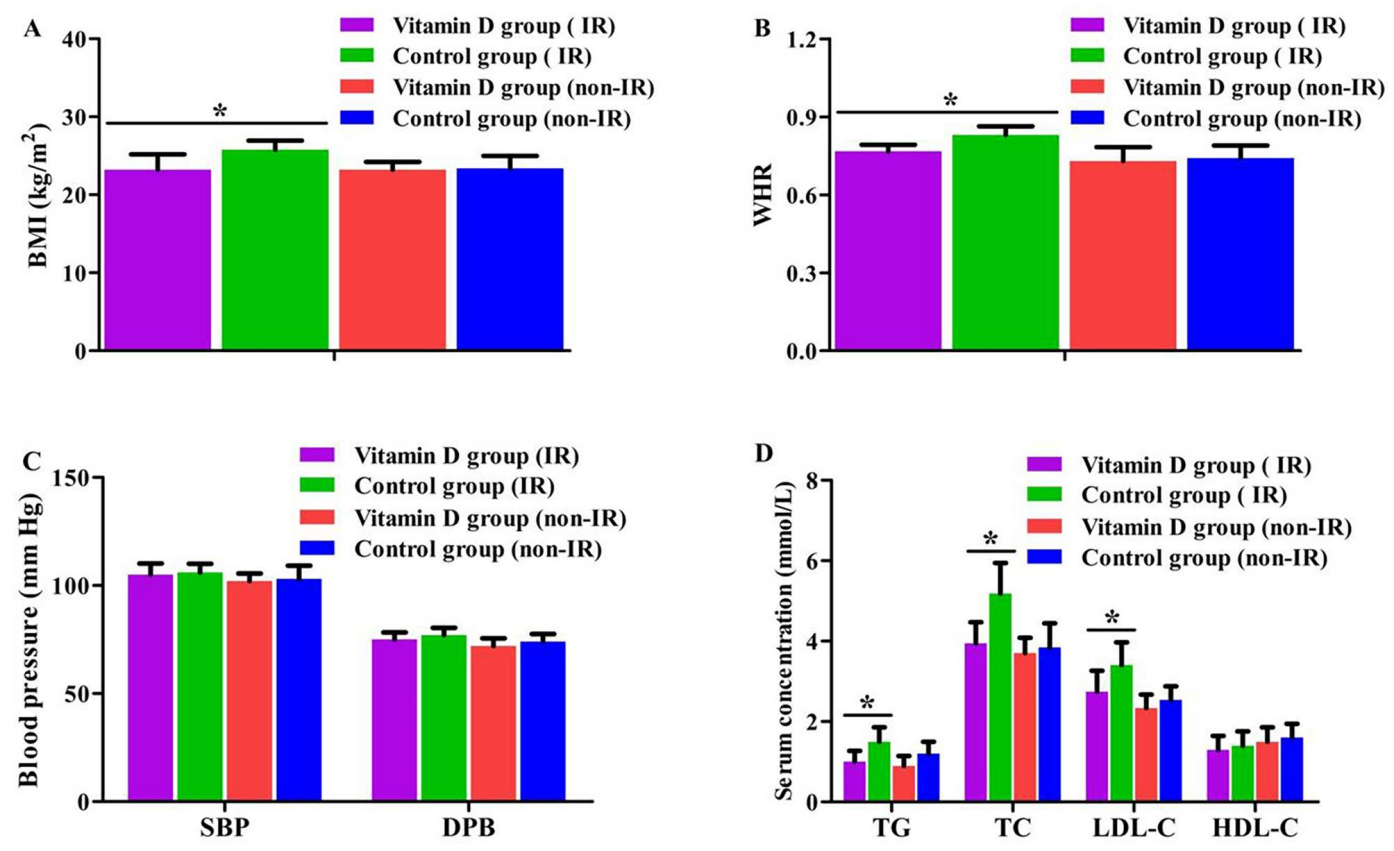
Comparison of metabolic parameters among the four groups (**A**: BMI, **B**: WHR, **C**: SBP and DPB, **D**: Blood lipid parameters. ^*^P < 0.05)

In addition, all participants were divided into four groups according to HOMA-IR: vitamin D group (IR) (*n* = 17), vitamin D group (non-IR) (*n* = 11), control group (IR) (*n* = 19), control group (non-IR) (*n* = 10). The BMI, WHR, TG concentration, TC concentration and LDL-C concentration in women of vitamin D group (IR) were significantly lower than that in control group (IR) (*P* = 0.022–0.047). However, no significant difference was seen observed when comparing the above parameters between vitamin D group (non-IR) and control group (non-IR) (*P* = 0.257–0.913). In addition, no significant difference was observed in SBP and DPB among the four groups (*P* = 0.897, *P* = 0.926) (Fig. 4).

## Discussion

The data in our study demonstrated that significant increases in vitamin D concentrations were shown at different time points after vitamin D supplementation. Furthermore, with the extension of treatment time, the serum 25(OH)D concentration gradually increased. The BMI, WHR, serum insulin concentrations and HOMA-IR in women of vitamin D group were significantly lower than that in control group. Although IR is not within any of the diagnostic criteria of PCOS in different countries, it occurs in most obesity and overweight women with PCOS, who have a form of IR intrinsic and the compensatory hyperinsulinaemia drives many of the phenotypic features of PCOS. Additionally, compared with lean PCOS women, women with obesity are more likely to accompany IR. In fact, obesity and IR interact with each other and form a vicious cycle, which are also difficult to treat clinically. Vitamin D dramatically improves glucose metabolism by increasing insulin production, insulin receptor expression and reducing pro-inflammatory cytokines [24]. Findings from the current study revealed that the serum insulin concentrations and HOMA-IR at different time points of OGTT in women of vitamin D group (obesity) were significantly lower than that in control group (obesity). Moreover, the BMI, WHR, TG concentration, TC concentration and LDL-C concentration in women of vitamin D group (IR) were significantly lower than that in control group (IR). Nevertheless, no significant difference was seen in metabolic parameters between vitamin D group (non-obesity) and control group (non-obesity), as well as between vitamin D group (non-IR) and control group (non-IR). A systematic review and meta-analysis of RCT reported that PCOS women with continuous low dose of vitamin D supplementation improve fasting glucose concentration and HOMA-IR, but the meta-analysis included studies of vitamin D in combination with other micronutrients [25]. Similarly, Menichini et al. confirmed that vitamin D supplementation (4000 IU/day) for a period of at least 12 weeks lead to improvement in terms of glucose level, insulin sensitivity, hyperlipidemia, and hormonal functionality in PCOS women [26].

The mechanism for vitamin D improving IR includes the following aspects: (a) Vitamin D receptor (VDR) is a key modulator of inflammation and β cell survival, whihc restored β cell function and ameliorate hyperglycemia in murine T2D models [27]. (b) Vitamin D increases insulin responsiveness for glucose transport through the binding of 1,25(OH)2D-VDR complex to the vitamin D response element of the insulin receptor [28]. (c) Vitamin D regulates the extracellular and intracellular calcium concentration, which is important for the mediation of glucose transport in the target tissues [29]. Nevertheless, Trummer et al. reported that Vitamin D supplementation had no significant effect on metabolic and endocrine parameters in PCOS [19]. Similarly, Ardabili et al. displayed that the fasting serum insulin and glucose levels, the insulin sensitivity and HOMA-IR did not change significantly by the end of the study [30]. The different results may be explained by different types of studies, dose and time of vitamin D supplementation, treatment with vitamin D alone or with other micronutrients, participants with pre-processed or not, lifestyles of the participants, place of residence and so on.

Our findings demonstrated that compared to control group, vitamin D supplementation significantly improved dyslipidemia in PCOS women with vitamin D deficiency and insufficiency, including reduced the serum TG, TC and LDL-C concentrations. Additionally, the serum TG, TC and LDL-C concentrations in women of vitamin D group (obesity) were significantly lower than that in control group (obesity). Similarly, some studies shown that vitamin D supplementation plus Calcium for eight weeks among vitamin D deficient women with PCOS had beneficial effects on serum TG and VLDL-cholesterol levels, but it did not affect other lipid profiles [31]. Sterol regulatory element-binding proteins (SREBPs) are transcription factors that control lipid homeostasis. Asano et al. screened a chemical library of endogenous molecules and identified 25-hydroxyvitamin D (25OHD) as an inhibitor of SREBPs activation. They found that vitamin D may regulate lipoprotein lipase gene expression and therefore, might decrease serum TC concentration [32]. However, the beneficial effect of vitamin D supplementation on lipid profiles was not found in other studies [33]. Different study designs and dosages of vitamin D supplementation, baseline data of participants might provide explanation for different results. Several mechanisms can explain the effects of vitamin D supplementation on serum TG and VLDL-cholesterol levels. 1,25-dihydroxy-cholecalciferol represses the expression of the apolipoprotein A-I (apo A-I) gene in hepatocytes, and vitamin D receptor modulators in hepatocytes and intestinal cells differentially regulate expression of the apo A-I gene [34]. In addition, the increased intracellular Calcium due to vitamin D supplementation in liver leads to stimulating microsomal triglycerides transfer protein (MTP), which is implicated in the formation and secretion of VLDL, and then results in decreased serum TG and VLDL-cholesterol levels [35]. However, the effect of vitamin D supplementation on lipid profiles in PCOS patients and its specific mechanism still needs further exploration.

Some limitations must be considered in this study. First, this was a single-centre RCT study in the city of Xi’an in Shaanxi, China. Second, androgen metabolic parameters were not observed in this study, including total testosterone, sex hormone-binding globulin and free androgen index, these parameters are currently under study by our team. Third, the effects of different doses of vitamin D supplementation on metabolic parameters in women with PCOS are not observed in this study, which will be explored in the future. Therefore, future studies are required to confirm the effectiveness of vitamin D on metabolic parameters of PCOS women, and the specific regulation mechanism are also need to be further explored.

## Conclusion

This study provides evidence that vitamin D supplementation significantly increased serum vitamin D concentration in PCOS women with vitamin D deficiency and insufficiency. Furthermore, this RCT study supports beneficial effects of vitamin D supplementation on metabolic parameters in PCOS women, including significant improvements in BMI, WHR, serum insulin concentrations and HOMA-IR, lipid metabolism parameters, especially in women with obesity or IR.

## Data Availability

All datasets generated for this study are included in the article. Further inquiries can be directed to the corresponding author.

## References

[1] Teede HJ, Tay CT, Laven JJE (2023). Recommendations from the 2023 international evidence-based guideline for the assessment and management of polycystic ovary syndrome. J Clin Endocrinol Metab.

[2] Guney G, Taşkın MI, Sener N (2022). The role of ERK-1 and ERK-2 gene polymorphisms in PCOS pathogenesis. Reprod Biol Endocrinol.

[3] Benham JL, Goldberg A, Teede H (2024). Polycystic ovary syndrome: associations with cardiovascular disease. Climacteric.

[4] Wimalawansa SJ (2018). Associations of vitamin D with insulin resistance, obesity, type 2 diabetes, and metabolic syndrome. J Steroid Biochem Mol Biol.

[5] Liu Y, Fan HY, Hu JQ (2023). Effectiveness and safety of acupuncture for insulin resistance in women with polycystic ovary syndrome: a systematic review and meta-analysis. Heliyon.

[6] Afandak F, Aryaeian N, Kashanian M (2023). Effect of sumac powder on clinical symptoms, hyperandrogenism, inflammation, blood glucose, lipid profiles in women with polycystic ovary syndrome: a double-blind randomized clinical trial. Phytother Res.

[7] Paschou SA, Polyzos SA, Anagnostis P (2020). Nonalcoholic fatty liver disease in women with polycystic ovary syndrome. Endocrine.

[8] Osibogun O, Ogunmoroti O, Michos ED (2020). Polycystic ovary syndrome and cardiometabolic risk: opportunities for cardiovascular disease prevention. Trends Cardiovasc Med.

[9] Szymczak-Pajor I, Sliwinska A (2019). Analysis of association between vitamin D deficiency and insulin resistance. Nutrients.

[10] Cojic M, Kocic R, Klisic A (2021). The effects of vitamin D supplementation on metabolic and oxidative stress markers in patients with type 2 diabetes: a 6-month follow up randomized controlled study. Front Endocrino.

[11] Musazadeh V, Kavyani Z, Mirhosseini N (2023). Effect of vitamin D supplementation on type 2 diabetes biomarkers: an umbrella of interventional meta-analyses. Diabetol Metab Syndr.

[12] Wang L, Lv S, Li F (2020). Vitamin D deficiency is associated with metabolic risk factors in women with polycystic ovary syndrome: a cross-sectional study in Shaanxi China. Front Endocrinol.

[13] Gokosmanoglu F, Onmez A, Ergenç H (2020). The relationship between vitamin D deficiency and polycystic ovary syndrome. Afr Health Sci.

[14] Zhang N, Liao Y, Zhao H (2023). Polycystic ovary syndrome and 25-hydroxyvitamin D: a bidirectional two-sample mendelian randomization study. Front Endocrinol.

[15] Tokmak A, Guzel AI, Güney G (2017). Effect of obesity on clinical parameters and pregnancy rates in women with polycystic ovary syndrome undergoing ovulation induction cycles. J Reprod Med.

[16] He C, Lin Z, Robb SW (2015). Serum vitamin D levels and polycystic ovary syndrome: a systematic review and meta-analysis. Nutrients.

[17] Javed Z, Papageorgiou M, Deshmukh H (2019). A randomized controlled trial of vitamin D supplementation on cardiovascular risk factors, hormones, and liver markers in women with polycystic ovary syndrome. Nutrients.

[18] Pittas AG, Jorde R, Kawahara T (2020). Vitamin D supplementation for prevention of type 2 diabetes mellitus: to D or not to D?. J Clin Endocrinol Metab.

[19] Trummer C, Schwetz V, Kollmann M (2019). Effects of vitamin D supplementation on metabolic and endocrine parameters in PCOS: a randomized-controlled trial. Eur J Nutr.

[20] Chen ZJ, Shi Y, Sun Y (2016). Fresh versus frozen embryos for infertility in the polycystic ovary syndrome. N Engl J Med.

[21] Jeanes YM, Reeves S (2017). Metabolic consequences of obesity and insulin resistance in polycystic ovary syndrome: diagnostic and methodological challenges. Nutr Res Rev.

[22] Esmaeili SA, Mohammadian S, Radbakhsh S (2019). Evaluation of vitamin D3 deficiency: a population-based study in northeastern Iran. J Cell Biochem.

[23] Zhao H, Zhang J, Cheng X (2023). Insulin resistance in polycystic ovary syndrome across various tissues: an updated review of pathogenesis, evaluation, and treatment. J Ovarian Res.

[24] Mohan A, Haider R, Fakhor H (2023). Vitamin D and polycystic ovary syndrome (PCOS): a review. Ann Med Surg.

[25] Lagowska K, Bajerska J, Jamka M (2018). The role of vitamin D oral supplementation in insulin resistance in women with polycystic ovary syndrome: a systematic review and meta-analysis of randomized controlled trials. Nutrients.

[26] Menichini D, Facchinetti F (2020). Effects of vitamin D supplementation in women with polycystic ovary syndrome: a review. Gynecol Endocrinol.

[27] Wei Z, Yoshihara E, He N (2018). Vitamin D switches BAF complexes to protect β cells. Cell.

[28] Dong B, Zhou Y, Wang W (2020). Vitamin D receptor activation in liver macrophages ameliorates hepatic inflammation, steatosis, and insulin resistance in mice. Hepatology.

[29] Gupta T, Rawat M, Gupta N (2017). Study of effect of vitamin D supplementation on the clinical, hormonal and metabolic profile of the PCOS women. J Obstet Gynaecol India.

[30] Ardabili HR, Gargari BP, Farzadi L (2012). Vitamin D supplementation has no effect on insulin resistance assessment in women with polycystic ovary syndrome and vitamin D deficiency. Nutr Res.

[31] Asemi Z, Foroozanfard F, Hashemi T (2015). Calcium plus vitamin D supplementation affects glucose metabolism and lipid concentrations in overweight and obese vitamin D deficient women with polycystic ovary syndrome. Clin Nutr.

[32] Asano L, Watanabe M, Ryoden Y (2017). Vitamin D metabolite, 25-Hydroxyvitamin D, regulates lipid metabolism by inducing degradation of SREBP/SCAP. Cell Chem Biol.

[33] Zhu W, Cai D, Wang Y (2013). Calcium plus vitamin D3 supplementation facilitated fat loss in overweight and obese college students with very-low calcium consumption: a randomized controlled trial. Nutr J.

[34] Wehmeier KR, Mazza A, Hachem S (2008). Differential regulation of apolipoprotein A-I gene expression by vitamin D receptor modulators. Biochim Biophys Acta.

[35] Cho HJ, Kang HC, Choi SA (2005). The possible role of Ca2^+^ on the activation of microsomal triglyceride transfer protein in rat hepatocytes. Biol Pharm Bull.

